# Localization and symbiotic status of probiotics in the coral holobiont

**DOI:** 10.1128/msystems.00261-24

**Published:** 2024-04-12

**Authors:** P. M. Cardoso, L. J. Hill, H. D. M. Villela, C. L. S. Vilela, J. M. Assis, P. M. Rosado, J. G. Rosado, M. A. Chacon, M. E. Majzoub, G. A. S. Duarte, T. Thomas, R. S. Peixoto

**Affiliations:** 1 Red Sea Research Center, Biological and Environmental Science and Engineering Division, King Abdullah University of Science and Technology, Thuwal, Saudi Arabia; 2 Laboratory of Molecular Microbial Ecology, Institute of Microbiology Paulo de Góes, Federal University of Rio de Janeiro, Rio de Janeiro, Brazil; 3 Center for Marine Science and Innovation; School of Biological, Earth and Environmental Sciences, The University of New South Wales, Sydney, New South Wales, Australia; 4 Computational Biology Center, King Abdullah University of Science and Technology (KAUST), Thuwal, Saudi Arabia; 5 Marine Science and Bioscience Programs, Biological, Environmental and Engineering Sciences Division, King Abdullah University of Science and Technology (KAUST), Thuwal, Saudi Arabia; Universiteit Leiden, Leiden, the Netherlands

**Keywords:** coral, probiotics, BMC, FISH, coral-associated microbes, tissue, location, *Halomonas* sp., Cobetia sp.

## Abstract

Corals establish symbiotic relationships with microorganisms, especially endosymbiotic photosynthetic algae. Although other microbes have been commonly detected in coral tissues, their identity and beneficial functions for their host are unclear. Here, we confirm the beneficial outcomes of the inoculation of bacteria selected as probiotics and use fluorescence *in situ* hybridization (FISH) to define their localization in the coral *Pocillopora damicornis*. Our results show the first evidence of the inherent presence of *Halomonas* sp. and *Cobetia* sp. in native coral tissues, even before their inoculation. Furthermore, the relative enrichment of these coral tissue-associated bacteria through their inoculation in corals correlates with health improvements, such as increases in photosynthetic potential, and productivity. Our study suggests the symbiotic status of *Halomonas* sp. and *Cobetia* sp. in corals by indicating their localization within coral gastrodermis and epidermis and correlating their increased relative abundance through active inoculation with beneficial outcomes for the holobiont. This knowledge is crucial to facilitate the screening and application of probiotics that may not be transient members of the coral microbiome.

**IMPORTANCE:**

Despite the promising results indicating the beneficial outcomes associated with the application of probiotics in corals and some scarce knowledge regarding the identity of bacterial cells found within the coral tissue, the correlation between these two aspects is still missing. This gap limits our understanding of the actual diversity of coral-associated bacteria and whether these symbionts are beneficial. Some researchers, for example, have been suggesting that probiotic screening should only focus on the very few known tissue-associated bacteria, such as *Endozoicomonas* sp., assuming that the currently tested probiotics are not tissue-associated. Here, we provide specific FISH probes for *Halomonas* sp. and *Cobetia* sp., expand our knowledge of the identity of coral-associated bacteria and confirm the probiotic status of the tested probiotics. The presence of these beneficial microorganisms for corals (BMCs) inside host tissues and gastric cavities also supports the notion that direct interactions with the host may underpin their probiotic role. This is a new breakthrough; these results argue against the possibility that the positive effects of BMCs are due to factors that are not related to a direct symbiotic interaction, for example, that the host simply feeds on inoculated bacteria or that the bacteria change the water quality.

## INTRODUCTION

Tropical coral reefs are among the most diverse ecosystems in the world (1), and critical to corals, the main engineering species of these environments, is the endosymbiosis with algae of the family Symbiodiniaceae, which is responsible for satisfying most of the coral’s energetic demands through photosynthesis (2). The loss of these endosymbionts from tissues, known as bleaching, is a common response to stress and is believed to be mostly correlated with oxidative stress (3, 4) and/or nutrient imbalance (5–8) in coral holobionts exposed to extensive heat anomalies. Increases in sea surface temperatures have caused extreme coral mortality by bleaching, devastating approximately 30% of the corals around the world (9) and creating an urgent need to pursue science-based measures for reef protection (10).

The algae endosymbionts, however, are not the only symbionts associated with corals, as these animals can harbor a multitude of microorganisms, such as fungi, bacteria, archaea, and viruses (11, 12). Symbiotic microbial communities support the functioning of most living organisms through different beneficial mechanisms, such as increasing input of nutrients, protection against pathogens, removal of toxic compounds, and support in reproduction and early development (13). Coral-associated microorganisms, more specifically, can inhibit the proliferation of pathogens (14), provide nutrients to their host (15, 16), remove harmful reactive oxygen species (ROS) (17) excessive DMSP (18), or hydrocarbons (19, 20), and participate in larvae settlement (21, 22).

The term beneficial microorganism for corals (BMC) has been coined to describe microorganisms with specific beneficial functions, and their manipulation has been proposed as a method to protect coral health and microbiome homeostasis in impacted environments (12). The inoculation of BMC consortia as probiotics has been demonstrated to protect corals against heat stress (18, 23), pathogens (23, 24), and oil pollution (19, 20); to improve coral growth through calcification (25, 26); and to facilitate recovery from bleaching (18, 27).

Research on coral probiotics is however still in its early stages. Although different bacterial functions have been hypothesized to be beneficial to the coral, little is known about where the microbial strains used as probiotics are localized within the host. In fact, although microbial cells and aggregates have been commonly found in coral tissues (28–32), their taxonomic identity and function are yet to be fully explored (31). Most studies on these associated microbes have focused on detecting a few bacterial groups, such as the genus *Endozoicomonas*, which is commonly and widely associated with corals (32), *Ralstonia* sp., Proteobacteria, and Actinobacteria (29, 30), which have been hypothesized to have roles such as phosphorus cycling and nutrient acquisition inside coral tissues. Furthermore, although bacterial isolates tested for BMC traits have been obtained from coral extracts or seawater surrounding corals, the strains that form part of the probiotic consortia have not been proven to be truly symbiotic to the corals.

Here, we address this knowledge gap by defining the localization of the members of a previously established probiotic consortium (23) within the coral species *Pocillopora damicornis. P. damicornis* is one of the most widespread species of reef-forming corals. Their fast-growing colonies can be found in multiple regions of the Indo-Pacific in a high variety of habitats (33), turning these important reef builders into important candidates to test coral probiotics. We further confirm the beneficial role of this consortium when delivered to fragments of *P. damicornis* in a heat stress experiment, also revealing additional physiological improvements that had not been tested before (i.e., productivity and calcification). Most importantly, our results show the first evidence of the inherent presence of *Cobetia* sp. and *Halomonas* sp. in the coral tissue (gastrodermis and epidermis) and that their enrichment correlates with improvement of the health of the holobiont under temperature stress.

## MATERIALS AND METHODS

### Mesocosm design and coral maintenance

The experimental mesocosm consisted of 20 2.3 L aquaria placed into four different water baths (100 × 50 × 10 cm) to maintain temperature (Fig. S1). Each aquarium was connected individually to a 9 L circulation sump, and water was circulated at a rate of 170 L h^−1^. The aquaria and sumps were filled with 10 L of natural seawater from the Marine Aquarium of Rio de Janeiro (AquaRio). The seawater used was treated by ozonation and sand filtered. A water exchange of 20% was performed every 3 days with fresh seawater. The water was bubbled in each aquarium and sump through hoses connected to an air pump (HG-370, Sunsun, JIangyin, China). The temperature of each water bath was controlled through two MT-518Ri thermostats (Full-Gauge, Canoas, Brazil). The thermostats were connected to a 25W aquarium heater (Hopar, Kraainem, Belgium) and a Sb2000 pump (Sarlo Better, São Caetano do Sul, Brazil) for cooling. The heaters were installed inside the water bath, whereas the pumps were installed in 290 L plastic containers filled with fresh water, alongside a 1.8 HP chiller (MundoSub, Itaboraí, Brazil) for cooling purposes. When activated, the pumps from the cooling system delivered water from a tank at 18°C at a rate of 1950 L h^−1^. The experimental aquaria were each illuminated by an LED fixture consisting of blue and white diodes individually controlled by a dimmer system. The light followed a 12:12 h cycle, with an intensity of 150 µmol of photons m^−2^s^−1^ from 9 AM to 3 PM and of 100 m^−2^s^−1^ from 6 AM to 9 AM and from 3 PM to 6 PM. Light intensity was verified with a Mq-210 light sensor (Apogee Instruments, Utah, USA). The water salinity, pH, and dissolved oxygen (DO) were measured in each aquarium every 2 days with a YSI Professional Plus instrument (YSI, Ohio, USA) and an InLab OptiOX DO sensor (Mettler Toledo, Ohio, USA). Salinity was corrected to 37 PSU with deionized water.

### Mesocosm experiment

**Fig 1:**
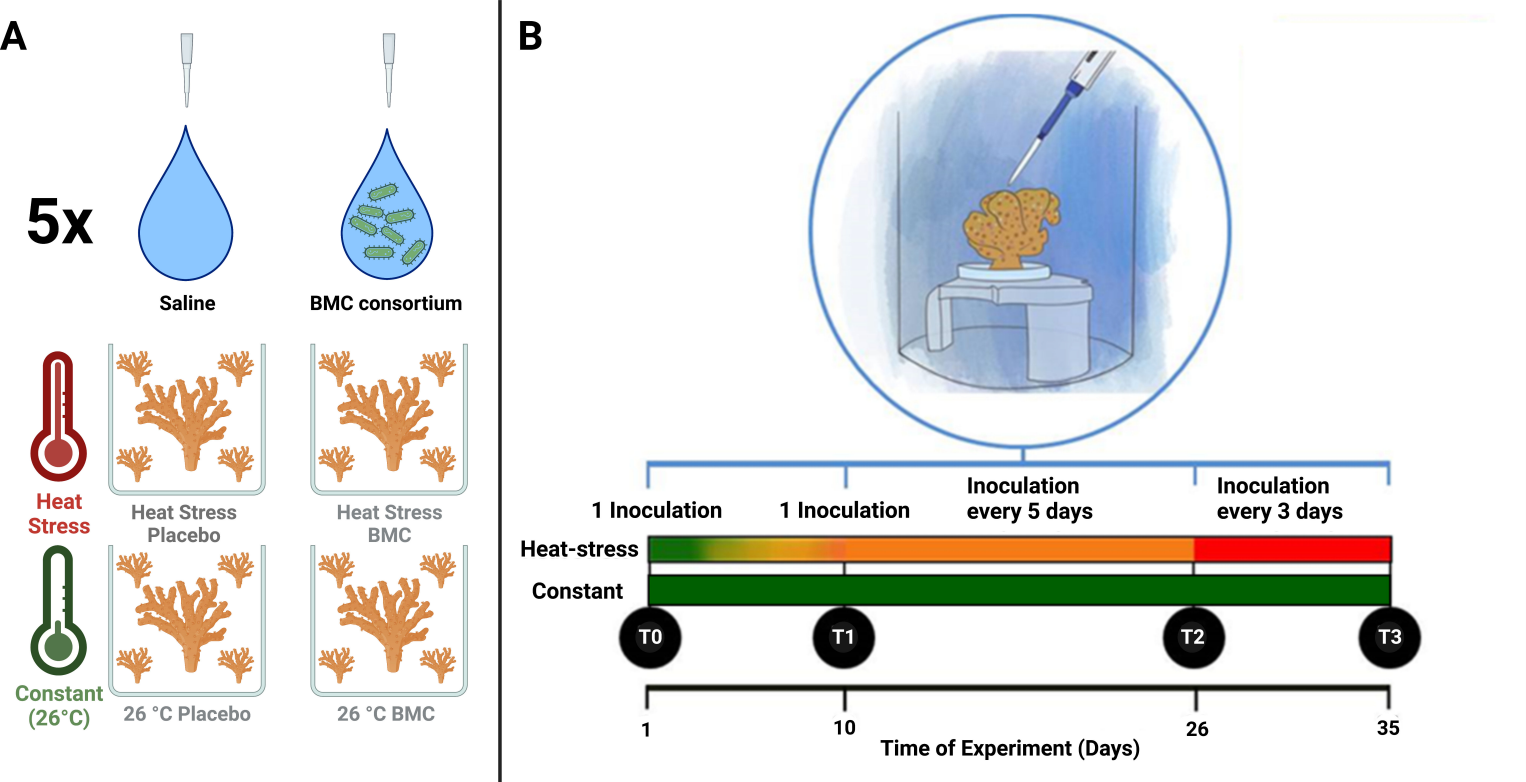
(**A**) Experimental design for temperature treatment and inoculation group. (**B**) Mesocosm experimental design, detailing inoculation frequency and temperature regime.

Three coral colonies of the species *P. damicornis* were purchased from the Piscicultura Tanganyika coral farm (Aquiraz, Ceará, Brazil) and maintained in a single aquarium for 34 days before being divided into 96 fragments with an approximate height of 3 cm. Four randomly selected fragments were placed in each aquarium for sampling. The fragments were acclimated for 18 days at 26°C before the beginning of the experiment. During the experiment, the aquaria were subjected to two temperature regimes (control temperature and heat stress) and two different inoculation treatments (placebo and BMCs) in a fully crossed design, with five replicate aquaria (Fig. 1A). Four of the five replicate aquaria had an extra fragment used for respiration, calcification, and photosynthesis assays. The corals were not fed during the acclimation period or the experiment.

The temperature regime of the control was maintained at 26°C throughout the experiment, whereas for the heat stress regime, an increase of 0.5°C each day until the temperature reached 30°C on the 10th day was applied. The temperature was then maintained at 30°C until the 26th day of the experiment and subsequently increased to 31°C until the 35th experiment day in order to induce an even higher temperature stress (Fig. S2), since the fragments obtained were from the Indo-pacific and are usually unstressed at 26°C, whereas heat-stress can already start to be observed at 30°C (34, 35).

For the inoculations, coral fragments were removed from the aquarium, placed into sterile petri dishes, where the bacterial consortium or saline solution was applied, and then immediately returned to the aquarium. The petri dishes were subsequently rinsed with sterile saline solution (0.85% NaCl), which was poured into the aquarium. For the placebo treatment, 1 mL of a sterile saline solution (0.85% NaCl) was applied, whereas 1 mL of a cell suspension in saline solution with 10^7^ cells of probiotics was used in the BMC treatment (see below). The use of a placebo as a negative (and therefore inert) control is the gold standard for probiotic validations, as there is no current way to rule out bacterial cells as non-BMC (due to the lack of knowledge about all possible BMC mechanisms), and the use of dead cells could infer confounding factors since dead cells may act as postbiotics and trigger specific host responses (36). Inoculations were performed on the first day of the experiment (T0), on the 10th day of the experiment (T1), every 5 days between T1 and T2, and every 3 days between T2 and T3 (Fig. 1B; Fig. S2). The intensified inoculation regime was an attempt to counteract the effects triggered by the increased temperature exposure with an intensified inoculation frequency.

Sampling occurred at four timepoints: T0 (day 1, before heat stress), T1 (day 10, after gradual temperature increase), T2 (day 26, after heat stress), and T3 (day 35, after increased heat stress). Collected coral replicates were cut into smaller fragments with sterile pliers and used for different analyses. Pieces used for nucleic acid extractions were placed in cryotubes, immediately flash frozen in liquid nitrogen, and stored at −80°C. Pieces used for fluorescence *in situ* hybridization (FISH) were placed into tubes filled with 4% paraformaldehyde in 10 mM PBS. During T0, T1, and T2, sample collection preceded the inoculation. On the days when the water exchange coincided with the inoculation, the exchange was performed first. Sample collection was performed at least 48 h after the last inoculation at every timepoint, and inoculations would be delayed by one day if they fell within this time frame. A total of five replicates of each experimental group were collected at each timepoint, and four replicates of each group were used for incubations for physiological parameters.

### Bacterial inoculum preparation

For the preparation of the BMC consortium, seven bacterial strains previously isolated from *P. damicornis* colonies and surrounding seawater (23) were used. Five strains (BMC 1–5) were classified as *Pseudoalteromonas shioyasakiensis*, one strain (BMC 6) as *Cobetia* sp., and the last (BMC 7) as *Halomonas* sp. based on genomic data (37). The seven isolates were grown separately from 80% glycerol culture stocks inoculated into 20 mL of marine broth (MB) media and cultivated in an incubator at 26°C for 16 h. Subsequently, 1 mL of the culture was passed into 100 mL MB and cultivated in the same conditions for 28 h to reach a concentration of ~3.5  ×  10^6^ viable cells mL^–1^ as verified from CFU counts on marine agar (MA) plates. Cells were washed by centrifugation at 4,000 × *g* for 2 minutes, resuspended in sterile saline solution (0.85% NaCl) three times, and finally resuspended in sterile saline solution to reach a concentration of 10^7^ viable cells mL^–1^ as described by (23, 23).

### DNA extraction, library preparation, and 16S gene metabarcoding

To assess the coral microbiome composition, sequencing of bacterial 16S rRNA gene amplicons was performed. Frozen coral samples were crushed with a sterile mortar and pestle, and 0.5 g of the crushed coral was used for DNA extraction using the DNeasy PowerBiofilm Kit (Qiagen, Venio, Netherlands), in parallel with extraction blanks without the addition of samples to account for possible contaminants, following the instructions provided by the manufacturer. PCRs for extraction blanks showed no visible bands, and their sequencing shows very few reads, suggesting no risk of contamination of our biological samples. DNA concentration was assessed using the Qubit dsDNA High Sensitivity (HS) kit (Invitrogen, Massachusetts, USA). The primers with Illumina overhang adapter sequences, 341F (TCGTCGGCAGCGTCAGATGTGTATAAGAGACAGCCTACGGGNGGCWGCAG) and 785R (GTCTCGTGGGCTCGGAGATGTGTATAAGAGACAGGACTACHVGGGTATCTAATCC), were subsequently used to amplify the V3-V4 regions of the 16S rRNA gene. The reaction mixture (50 µL of total volume per sample) consisted of Econotaq PLUS GREEN 2X Master Mix (Lucigen) (25 µL), Ambion nuclease-free water (17 µL), the primer pair 341F and 785R (1.5 µL of each; 10 µM), and DNA template (5 µL). The first stage PCR program comprised an initial denaturation at 94°C (2 min), followed by 35 cycles of denaturation at 94°C (30 s), annealing at 55°C (30 s), extension at 72°C (40 s), and a final extension of 72°C (7 min). PCR products were then quantified using gel electrophoresis and cleaned using the QIAquick PCR Purification Kit (Qiagen, Hilden, Germany) following the manufacturer’s instructions. The cleaned products were then indexed through eight PCR cycles using the Nextera compatible i7 and i5 index primers (Illumina, San Diego, CA, USA) and the KAPA HiFi HotStart Readymix (Roche, Basel, Switzerland) as the PCR reagent. Paired-end sequencing (2 × 300 bp) of the resulting 16S rRNA gene amplicons was performed at the Ramaciotti Centre for Genomics (RCG), UNSW, on the Illumina MiSeq platform as per the MiSeq System User Guide (Illumina 2013).

Sequence data were initially quality‐filtered and trimmed using Trimmomatic version 0.36 (38), truncating reads if the quality dropped below 15 in a sliding window of 4 bp. USEARCH version 11.0.667 (39) was used for further processing as described by (40, 40) to merge sequencing reads and quality‐filter contigs, excluding sequences with <250 or >550 nucleotides, in addition to sequences with more than one ambiguous base or an expected error of more than 1. Filtered sequences were denoised and clustered into unique sequences (zero-distance operational taxonomic units[zOTUs]) using the UNOISE3 algorithm implemented in USEARCH. Chimeric sequences were removed with UCHIME *de novo* during zOTU clustering and subsequently with a reference-based comparison against the GTDB v202 database (41). zOTUs were taxonomically classified using a Bayesian Last Common Ancestor algorithm (BLCA) (42) against the GTDB v202 and SILVA v138 databases (43). All nonbacterial OTUs were removed along with singleton OTUs. Finally, the quality-filtered sequences were mapped on zOTU sequences to calculate the counts of each zOTU in every sample. zOTUs that were putatively contaminants were detected using the decontam package (44).

To investigate the presence of bacterial strains used as an inoculum, their 16S rRNA gene sequences (37) were aligned against the zOTUs obtained using BLASTn. zOTUs that had a 100% sequence similarity and coverage of the V3-V4 regions with the 16S rRNA gene sequences obtained from the isolates were considered the same bacterial strain used as an inoculum, and their relative abundance was compared between each experimental group.

The tests on the zOTU count and taxonomy tables were performed with the Phyloseq (45) and dplyr (46) packages in RStudio version 2022.12.0+353 (47). Taxonomic data previously normalized by relative abundance (percentage) was used for the calculation of dissimilarities based on the Bray-Curtis index, which was then visualized by principal coordinate analyses. Pairwise differences between each group were assessed with PERMANOVA. To study the alpha diversity, ASV data were rarefied with the Phyloseq “rarefy_even_depth” function and used for Shannon index calculations. The normality of this data was tested using the Shapiro-Wilk test. Differences between the groups were tested with analysis of variance (ANOVA) tests for timepoints with normal data and Wilcoxon tests for data that did not follow normality. To test for specific zOTUs that were indicators of each treatment, the “indicspecies” package (48) was used with read data not subjected to rarefaction, with a *P*-value cutoff of 0.05. All images and statistical tests based on the 16S rRNA gene amplicon data were created and performed using ggplot2 (49).

### Oligonucleotide probe design for fluorescence *in situ* hybridization

Previously obtained 16S rRNA gene sequences (37) were used for the design of oligonucleotide probes to specifically target the bacterial taxa used as coral probiotics. To achieve this, BLASTn searches against the NCBI database were used to find similar sequences from the strains that were targeted and other species with almost identical 16S rRNA genes. Target sequences, those obtained from the search results and the *Escherichia coli* 16S rRNA gene sequence, were then aligned using BioEdit software (50) to identify unique regions for the target and assess potential fluorescence based on the data from (51, 51). The candidate probe’s sequence was then tested in OligoCalc software (52) for self-complementarity and followed the criteria: size of 18–25 bps, GC content of 40% to 60%, and nearest neighbor Tm > 57°C (in the condition of salt and primer concentrations of 50 mM and 50 µM, respectively). Finally, the selected sequences were tested using RDP’s probematch (53) and Silva’s TestProbe (43) search tools to check for the highest possible specificity to target strains and confirm that probes only targeted the genera the strains belonged to. The resulting probe sequences were: HAL847 (targeting the *Halomonas* strain), 5' ATT GCG TTA ACT GCG CCA CTA AGA 3', and COB1268 (targeting the *Cobetia* strain), 5' AGC TTT ATG GGA TTG GCT CCA CGT 3'. All probes were labeled with the Alexafluor 532 fluorochrome on their 5’ end. Formamide stringency for each probe was also tested on target isolates through flow cytometry. Approximately 10^6^ cells were hybridized in 90 µL of buffer containing 0.9 M sodium chloride, 0.1% sodium dodecyl sulfate, 20 mM Tris-HCl (pH 7.2), and varying concentrations of formamide (10, 20, 30, 40, and 50%), with 10 µL of fluorescent probe solution (at a concentration of 25 ng/µL) at 46°C for 1.5 h. Subsequently, cells were centrifuged for 2 min at 4,000 × *g* and resuspended in 100 µL of hybridization buffer containing no probe. After incubating for 30 min at 46°C, the samples were mixed with 500 µL of 1× PBS and subsequently placed on ice before the analysis. Hybridization reactions with fixed cells were done in triplicates. After diluting the resulting cell suspension 100-fold in 1× PBS, the fluorescence intensities of hybridized cells were quantified with a CytoFLex flow cytometer (Beckman Coulter Life Sciences Biosciences, Brea, CA, USA), using the 488 nm emission line argon ion laser as the light source (Fig. S3) and the PE fluorescence filter. Normalized fluorescence median values were calculated as described in reference 54, using blanks without added cells to detect the background noise for the probes, and plotted against the theoretical formamide curve obtained for each probe from ProbeMelt software (54).

Additionally, probes were subsequently tested on target and nontarget bacterial isolates, using both *Cobetia* sp. and *Halomonas* sp. as specific targets for the probes and strains with two mismatches in relation to the probe sequences as nontarget isolates (Table S1). The *Cobetia sp*. strain used as a probiotic in this study was used to test the HAL847 probe, and the M24 *Bacillus oshimensis* strain (6) was hybridized with the COB1268 probe to test its specificity and hybridized with the EUB338 (5′ GCT GCC TCC CGT AGG AGT 3′) (55) eubacteria-specific probe in parallel as a positive control for FISH. The *Halomonas* sp. and *Cobetia* sp. BMC strains were also submitted to the same hybridization conditions with nonsense probes NON-EUB338 (5′ ACT CCT ACG GGA GGC AGC 3′) (56) to ensure that positive signals were specific. For this, we adapted the protocol developed by (57, 57): pure cultures of *Cobetia* sp., *Halomonas* sp., and *B. oshimensis* were fixed in parallel with 4% paraformaldehyde for 1 h, washed three times in 1× PBS and mounted on microscope slides. Samples were dehydrated in ethanol series (50%, 70%, and 100%) for 2 min each, embedded in a preheated (37°C) solution proteinase k (50 µg/mL) for 5 min, hybridized in 9 µL hybridization buffer (30% [vol/vol] formamide, 0.9 M NaCl, 20 mM Tris–HCl (pH 8.0), and 0.01% SDS), and 1 µL of the desired probe at a concentration of 25 ng/µL for 1 h 30 minat 46°C. Samples were washed with preheated (46°C) washing buffer (0.112 M NaCl, 20 mM Tris–HCl [pH 8.0], 0.01% SDS, and 5 mM EDTA) for 10 min, treated with a drop of DAPI solution (1 µg/mL), and rinsed with distilled water and finally mounted with Citifluor mounting media (EMS, Hatfield, USA). Cells were visualized using a Leica DM6000 B fluorescence microscope with the DAPI and Cy3 filters (Fig. S4).

### Coral histological preparation and fluorescence *in situ* hybridization

Tissue preparation and FISH followed protocols established by reference 58, maintaining the methodology for tissue fixation, storage, and decalcification while modifying the following steps: after the coral skeleton was decalcified, agarose embedded samples were rinsed with a 10 mM PBS solution and dehydrated sequentially with ethanol solutions for 15 min in each of the following concentrations: 30%, 50%, 70%, 90%, and 100% (two incubations were performed at 100%). The final 100% ethanol solution was exchanged for a 1:1 absolute ethanol and LR White (London Resin White, medium grade) mixture, in which the samples were kept for 12 h. Subsequently, the ethanol/resin solution was removed, and pure resin was applied. The samples soaked with resin were carefully tilted every 15 min, and after 1 h, the resin was exchanged for fresh resin; the process was repeated for another hour. After the second incubation in pure resin, the LR White was removed, and the tissue samples were transferred to BEEM capsules, which were filled with resin and allowed to polymerize under UV light at −20°C for 5 days. The resulting blocks were serially sectioned (1 µm) and used for FISH using the custom probes, as well as the eubacterial EUB338 and nonsense NON-EUB338 probes as positive and negative controls, with the Alexafluor 532 and 647 fluorophores attached, respectively. The histological sections were placed on glass slides previously coated with a 0.1% agarose and 0.01% KCr(SO_4_)_2_ and maintained at 4°C before the collection of the sections. Histological sample sections were then dehydrated through serial 5 min washes with ethanol at concentrations of 50%, 70%, and 100%, followed by air-drying. Dry sections were washed with a drop of 0.2 M HCl for 12 min, followed by a drop of Tris-HCl solution (pH 8.0) for 10 min at room temperature. Sections were dried with paper between the two washing solutions and after the Tris-HCl wash. The sections were then treated with proteinase K (50 µg/mL) diluted in Tris–HCl (pH 8.0) for 5 min at 37°C and rinsed in 20 mM Tris HCl (pH 8.0). For the hybridization, sections were treated with 8 µL of the hybridization buffer (30% [vol/vol] formamide, 0.9 M NaCl, 20 mM Tris–HCl [pH 8.0], 0.01% SDS), 1 µL of the specific oligonucleotide probe solution (i.e., either EUB338, HAL847, or COB1268), and 1 µL of the NON-EUB338 probe solution (to account for unspecific binding). Probe solutions were stocked at a concentration of 25 ng/µL. The sections were incubated in a humidity chamber for 1.5 h at 46°C and subsequently washed with a drop (~10 µL) of preheated washing buffer (0.112 M NaCl, 20 mM Tris–HCl [pH 8.0], 0.01% SDS, and 5 mM EDTA) for 10 min in a water bath at 48°C. Subsequently, a drop of distilled water was applied to sections for the removal of excess salt. Finally, the slides were mounted with a FluoroShield + DAPI mounting media (Sigma-Aldrich, Massachussets, USA). The slides were then visualized with a Leica SPE Confocal Laser Scanning microscope with an excitation laser set at 532 nm (for the Alexa Fluor 532), 635 nm (for the Alexa Fluor 647), and 405 nm (for DAPI) and emission bands of 540–560 nm (for Alexa Fluor 532), 640–680 nm (for Alexa Fluor 635) and 400–515 nm (for DAPI). All stack images used to create the panels (i.e., images from coral tissues) were deconvoluted and compensated to correct 3D fluorescent spectral spillovers using the Leica LAS AF software and processed using ImageJ (59) to build maximum intensity z-stack and 3D reconstruction videos, and specific lookup tables were attributed to each fluorescence channel for a better visualization.

### Coral bleaching and photosynthetic efficiency evaluation

The coloration of coral fragments was evaluated from photographs taken under standard conditions and compared with a color chart (60). The photosynthetic efficiency of coral endosymbiotic algae was assessed through Pulse-Amplitude Modulation (PAM) fluorometry with a diving-PAM instrument (Walz GmbH, Effeltrich, Germany). *F*_v_/*F*_m_ ratios were measured for each replicate fragment on multiple days throughout the experiment by placing the instrument’s fiber optic cable over one of the fragment’s polyps once the corals were dark-acclimated (2 h after the lights were off). The parameters used with the fluorometer were as follows: saturation pulse = 8, gain = 3, measuring light = 5, saturation light amplitude = 0.8, and damping = 2. Differences of *F*_v_/*F*_m_ data over time were tested by the creation of linear mixed effect models using the “lmer” function of the “lme4” package (61), treating replicates as a random effect on the model’s intercept. *F*_v_/*F*_m_ was treated as a response variable, time as a predictor variable, and treatment as a factor with four levels. Significant differences between treatments in relation to slopes were then tested using the “emtrends” function from the “emmeans” package with the Tukey test and kenward-roger degrees-of-freedom options (62).

### Coral calcification, respiration, and primary productivity assays

Incubations for physiological assays were performed in 250 mL acrylic chambers in water baths set to 26°C. Temperature was maintained using MT-518Ri thermostats (Full-Gauge, Canoas, Brazil) connected to heaters and an aquarium chiller. The water baths were connected to a tank containing the heaters and chiller, and water was pumped at a 1,950 L h^−1^ flow rate. The water from the chambers was agitated with HI190M magnetic stirrers (Hanna, Woonsocket, USA). For all incubations, seawater filtered with 0.22 µm pore filters was used, and the system was illuminated by fluorescent aquarium lights at an intensity of 100 photons m^−2^s^−1^. During incubations, four replicates from each treatment were used in parallel with four chambers filled with seawater as controls. For calcification, light-adapted corals were incubated for 3 h, and the initial and final seawater was collected for total alkalinity determination. The water was sterilized with 5 drops of chloroform for sample conservation. Samples were titrated with a Titrino 848 titrator for total alkalinity anomaly (63). For respiration and primary productivity, dissolved oxygen during incubations was measured with an InLab OptiOX DO sensor (Mettler Toledo, Ohio, USA). For net primary productivity, light-adapted corals were incubated for 2 h, whereas the respirometry incubations were performed with dark-adapted corals for 1 h. The difference between initial and final oxygen concentrations was used for calculations of respiration and net primary productivity. Gross primary productivity was calculated by adding the amount of oxygen from the gross primary productivity to the oxygen lost by the respirometry values (64). Alkalinity and oxygen values were normalized by the volume of water and the surface area of the fragments, determined by the paraffin single-dipping method as described by (65, 65). Physiological data were tested using a two-way PERMANOVA based on Euclidean distances for each timepoint using the dplyr package, with temperature and probiotics as factors. Samples were further compared using pairwise PERMANOVA between each group, applying Bonferroni corrections. The homogeneity of dispersion was tested through the “permtest” function in the vegan package. Data points that did not follow homoscedasticity were tested with a Scheirer–Ray–Hare test, followed by a pairwise Dunn test using the “BH” correction.

## RESULTS

### The microbiome of BMC-treated corals

Shifts in the coral microbiome related to heat stress and the inoculation of the BMC consortium were investigated through analysis of the V3-V4 region of the 16S rRNA gene. Of the 4,608 zOTUs generated, 23 were considered possible contaminants and are listed in Table S4. Significant differences were found in the Shannon indices of coral-associated bacterial assemblages from experimental treatment samples inoculated with a placebo under heat stress and at 26°C on the 26th day of the experiment (Wilcoxon test, *P* = 0.0079), although no statistical support for any pairwise differences were found in any of the other timepoints (Wilcoxon test, *P* > 0.05) (Fig. S5).

**Fig 2:**
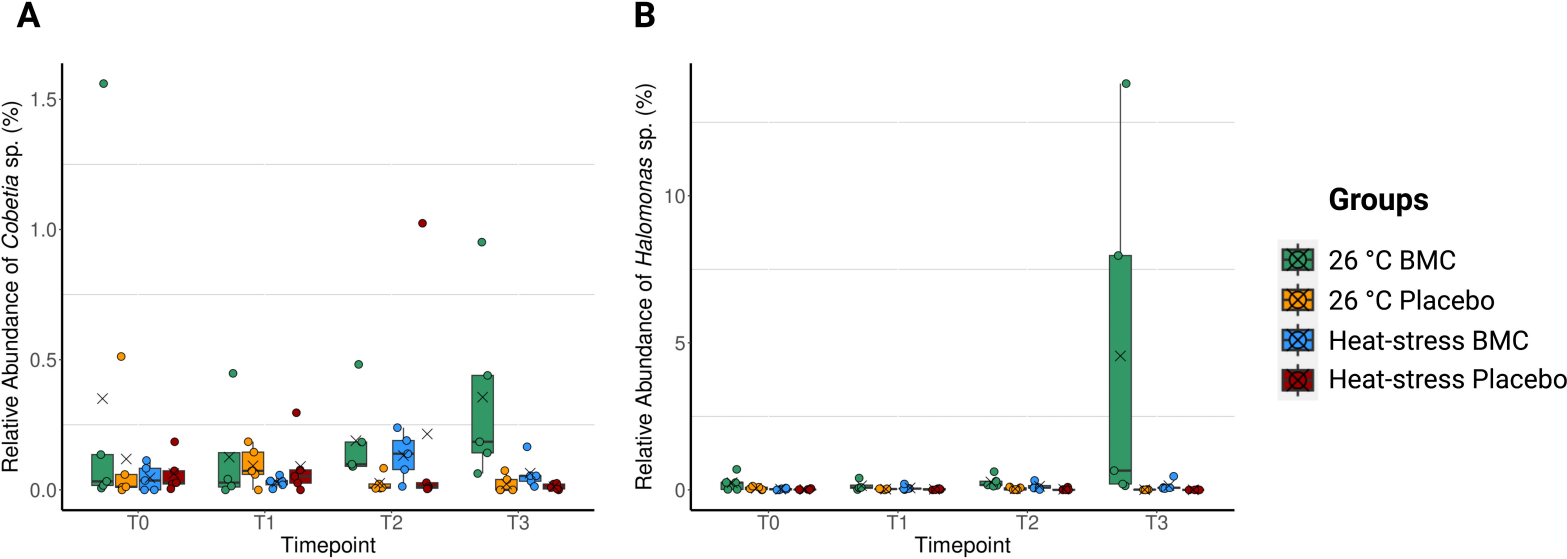
Relative abundances of *Cobetia* sp. (**A**) and *Halomonas* sp. (**B**) found in *P. damicornis* fragments, during the four timepoints of the experiment: T0 (day 1), T1 (day 10), T2 (day 26), and T3 (day 35), *n* = 5. Jitter points represent values from individual replicates, crosses represent the mean values of each group, and box edges represent quartiles, whereas middle horizontal bars represent median values.

The overall community structure of the coral microbiome was also similar throughout the experiment, with similar taxon profiles between treatments at each timepoint (Fig. S6) (pairwise PERMANOVA, Pr(>F) >0.05) and no evident separation of groups in ordination plots (Fig. S7); the only exception being significant differences between heat-exposed and constant-temperature BMC-treated samples on the 26th day of the experiment (pairwise PERMANOVA, Pr(>F) =0.03). Despite a general lack of major changes in the overall microbiome between most treatments, increases in the relative abundances of zOTUs related to the isolates used as probiotics were found on the 26th and 35th days of the experiment (T2 and T3) in inoculated samples. zOTU data did not show a clear pattern for *Pseudoalteromonas* sp. with inoculation, likely due to the abundance of this genus in samples. However, two members of the probiotic consortium, *Cobetia* sp. and *Halomonas* sp., had sequences with 100% identity to one zOTU each and were selected as indicator species of the probiotic treatment during T2 (*Halomonas* sp. *P* = 0.0079 in inoculated groups at 26°C; *Cobetia* sp. *P* = 0.0079 at 26°C) and T3 (*Halomonas* sp. *P* = 0.0075 at 26°C, *P* = 0.0092 at heat stress; *Cobetia* sp. *P* = 0.0151 at 26°C, *P* = 0.0346 at heat stress), which shows their increase in relative abundance through inoculation (Fig. S8). We therefore focused on the investigation of these two BMCs and the single zOTUs identical to their sequences (Fig. 2).

Inoculation was a significant factor in increasing the relative abundance of *Cobetia* sp. at T3 (Scheirer–Ray–Hare, *P* = 0.00186), and its increased relative abundance at T3 and 26°C was confirmed in inoculated corals by pairwise comparisons with uninoculated corals at the same temperature (Dunn’s multiple comparisons, adjusted *P* = 0.0123) (Fig. 2A). The increased abundance of *Halomonas* sp. in inoculated corals compared with placebo-treated corals was also confirmed, especially at 26°C, in both T2 (Scheirer–Ray–Hare, *P* = 0.00240) and T3 (PERMANOVA, R2 = 0.121, Pr(>F) =0.0001). Specifically, a significant increase in abundance of *Halomonas* sp. was observed when comparing BMC-treated samples with placebos at T2 at 26°C (Dunn’s multiple comparisons, adjusted *P* = 0.0378) and at both heat-exposure (pairwise PERMANOVA, *P* = 0.049) and 26°C (pairwise PERMANOVA, *P* = 0.049) during T3 (Fig. 2B).

### Localization of probiotic strains in coral tissues

**Fig 3:**
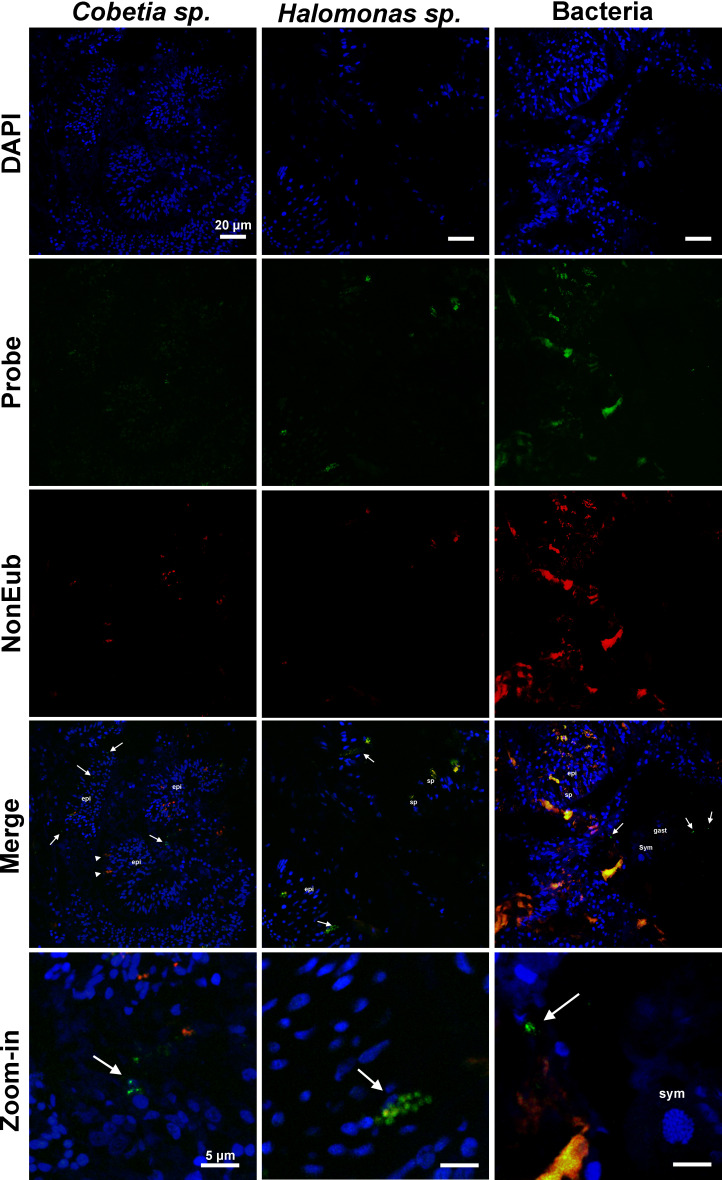
Fluorescence *in situ* hybridization images show the specific bacterial symbionts associated with tissue samples of *P. damicornis* of the experimental groups treated only with saline solution (placebo) and subjected to heat treatment on the 26th day of the experiment (T2). The first column shows samples hybridized with a probe specifically designed for *Cobetia* sp. (in green), the middle column shows samples hybridized with a probe specifically designed for *Halomonas* sp. (in green), and the last column shows samples hybridized with a general Eub-338 probe (in green). All hybridizations were performed with a NonEub-338 probe (in red) to account for unspecific staining, and all sections were also stained with DAPI (in blue). Sym = Symbiodiniaceae cells; epi = epidermis; gast = gastrodermis; sp = spyrocyst; arrowhead = granular gland cell; arrow = bacteria. Scale bars in first four lines = 20 µm, scale bars at last line = 5 µm.

**Fig 4:**
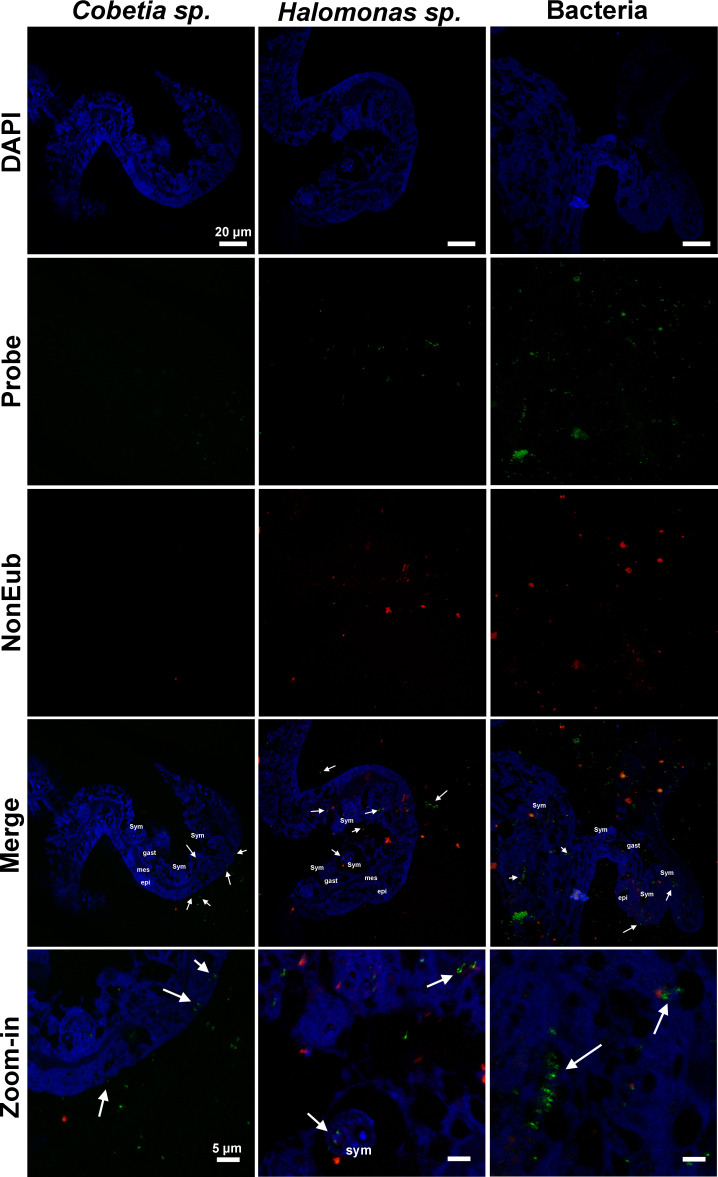
Fluorescence *in situ* hybridization images show the specific bacterial symbionts associated with tissue samples of *P. damicornis* incubated with BMC and subjected to heat treatment on the 26th day of the experiment (T2). The first column shows samples hybridized with a probe specifically designed for *Cobetia* sp. (in green), the middle column shows samples hybridized with a probe specifically designed for *Halomonas* sp. (in green), and the last column shows samples hybridized with a general Eub-338 probe (in green). All hybridizations were performed with a NonEub-338 probe (in red) to account for unspecific staining, and all sections were also stained with DAPI (in blue). Sym = Symbiodiniaceae cells; epi = epidermis; gast = gastrodermis; mes = mesoglea; arrowhead = bacterial aggregates; arrow = bacteria cells. Scale bars in first four lines = 20 µm, scale bars at last line = 5 µm.

FISH was performed with probes designed to specifically target the two bacterial strains enriched through probiotic inoculation. The goal was to investigate whether these enriched probiotics could be found associated with coral tissues, before or after their inoculation. Images obtained from coral tissue samples from heat-exposed placebo (Fig. 3) and BMC-treated (Fig. 4) groups on the 26th day of the experiment revealed the presence of bacteria hybridizing with the EUB338 probe, as well as fluorescent signals indicating the presence of *Halomonas* sp. and *Cobetia* sp. before and after their inoculation.

Images of heat-exposed coral tissues treated with placebo showed both *Halomonas* sp. and *Cobetia* sp. cells within the epidermal cells (Fig. 3), whereas images of the BMC-treated coral tissues showed these cells clearly within epidermal and gastrodermis cells, as well as around Symbiodiniaceae cells (Fig. 4). These results were also repeatedly found in 5–10 slides per treatment. NON-EUB338 probe showed regions of unspecific binding that typically corresponded to spirocysts and granular gland cells.

### Probiotic treatment affects key physiological processes

**Fig 5:**
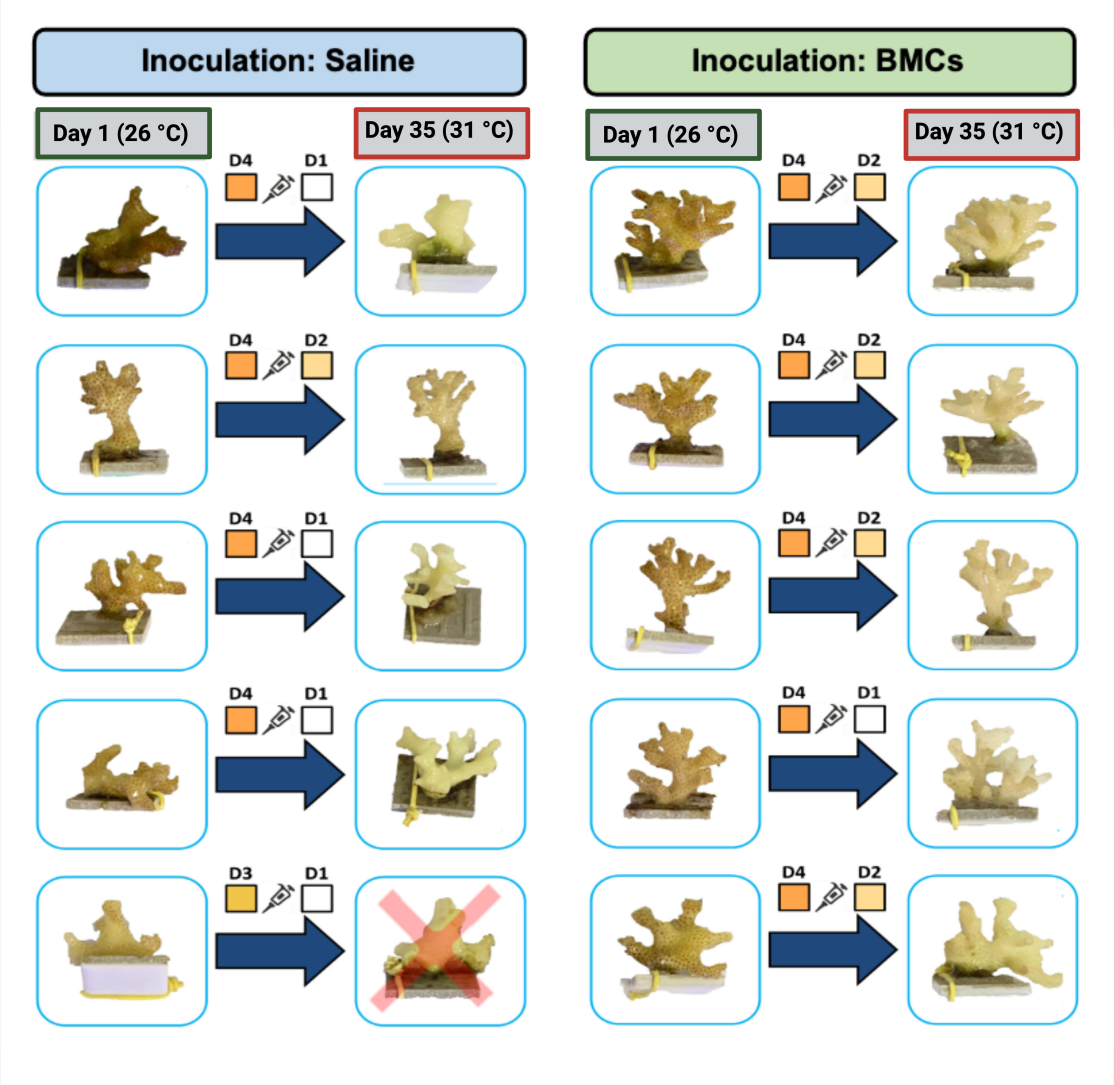
Comparative photographs of *P. damicornis* coral fragments used in the aquarium experiments before (day 1) and after (day 35) exposure to heat stress and treatment with the probiotic consortium. Colored squares indicate colors found in the coral chart for each replicate, and red crosses represent dead coral fragments.

Photographs taken throughout the experiment demonstrated that coral fragments in the constant temperature treatment (26°C, placebo or BMC-treated groups) maintained the same coloration levels until the end of the experiment. Fragments subjected to the heat stress treatment inoculated with saline solution (Placebo) had an average decrease of 2.6 degrees in the color scale, whereas fragments exposed to heat stress and inoculated with the probiotic consortium had an average decrease of 2 degrees (Fig. 5).

**Fig 6:**
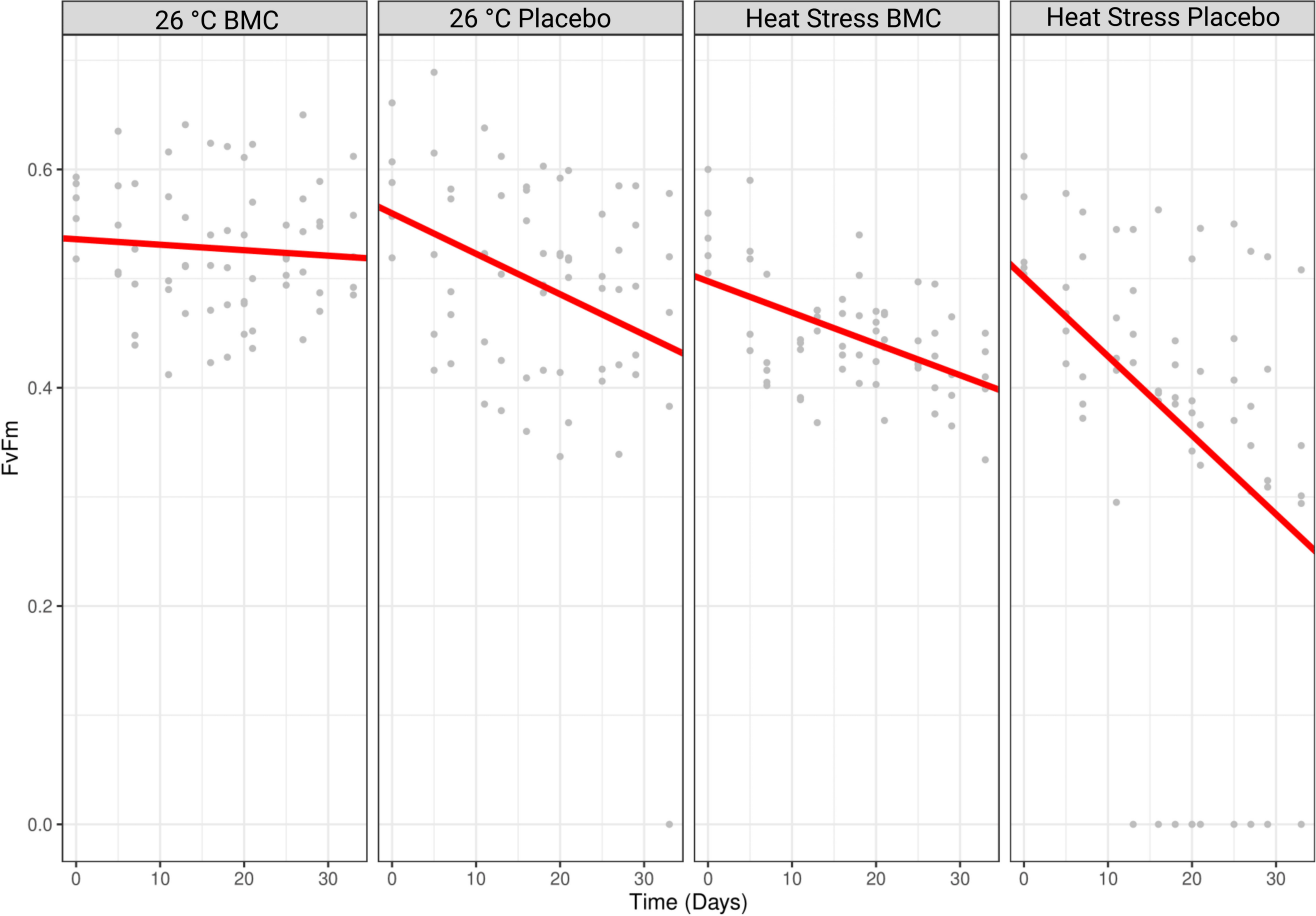
*F*_v_/*F*_m_ values of coral fragments treated with a placebo or the BMC consortium and exposed to heat stress or maintained at a constant temperature of 26°C (*n* = 5). Gray dots represent the specific *F*_v_/*F*_m_ values found for each replicate during different timepoints of the experiment. Red lines represent the fit of a mixed-effect linear model.

The photosynthetic potential of endosymbiotic algae measured by PAM fluorometry also changed by the end of the experiment. Comparing the first day of the experiment (day 1, T0) with the last (day 35, T3), placebo and BMC-treated corals maintained at constant temperature had a reduction in their Fv/Fm average values of 33.49 and 5.66%, respectively; instead, fragments of the heat-stressed samples that received a placebo or BMC had a reduction in 46.61 or 25.60% of their average values, respectively (Fig. 6). The reduction rates in Fv/Fm values over time significantly differed when comparing BMC-treated corals with fragments treated only with saline solution, both in the constant temperature (Tukey, *P* = 0.0153) and heat-stress (Tukey, *P* = 0.003) treatments.

**Fig 7:**
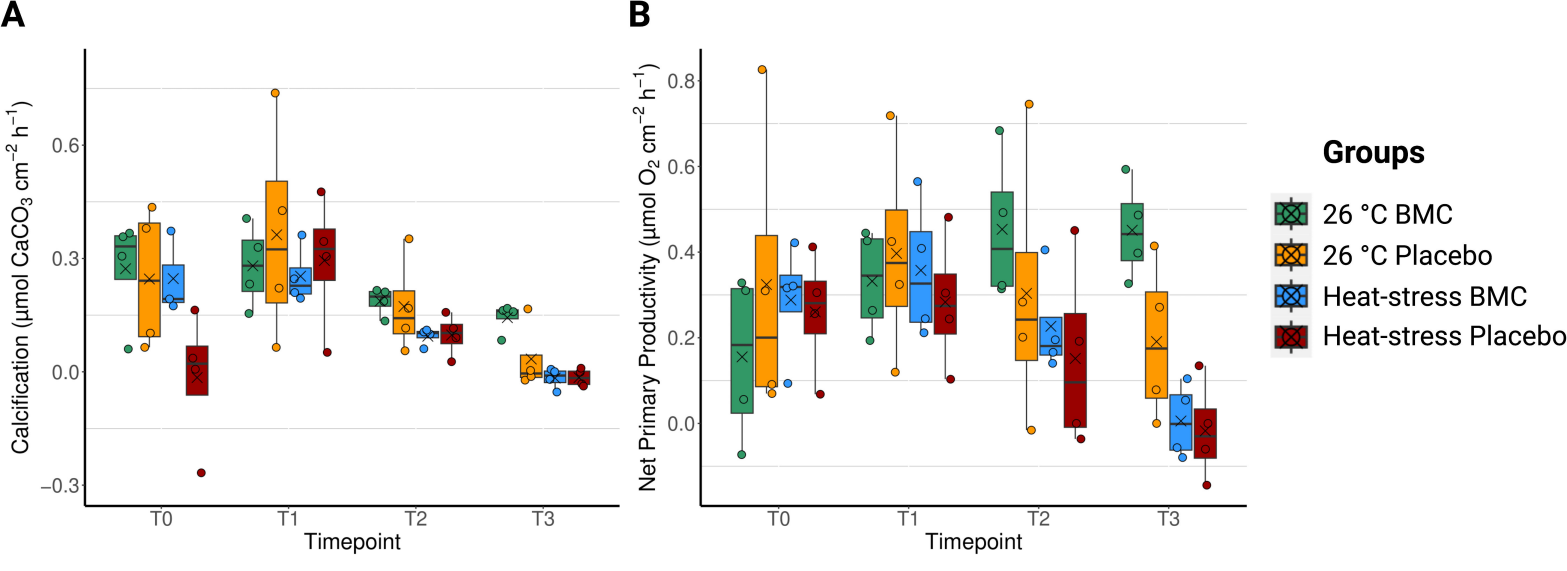
Calcification (**A**) and net primary productivity (**B**) values in *P. damicornis* fragments during the four timepoints of the experiment: T0 (day 1), T1 (day 10), T2 (day 26), and T3 (day 35), *n* = 4. Jitter points represent values from individual replicates, crosses represent mean values of each group, box edges represent quartiles, whereas middle horizontal bars represent median values.

To investigate the effect of the BMC treatment and temperature on coral physiology, incubations were performed to establish their photosynthetic, respiration, and calcification rates. No significant differences were found in calcification rates between experimental treatments in relation to the temperature regime (PERMANOVA, T0: R2 = 0.045, Pr(>F) =0.9264; T1: R2 = 0.021, Pr(>F) =0.6339), the inoculation treatment (PERMANOVA, T0: R2 = 0.088, Pr(>F) =0.076; T1: R2 = 0.036, Pr(>F) =0.5269), or the interaction between the two factors (PERMANOVA, T0: R2 = 0.083, Pr(>F) =0.1226; T1: R2 = 0.004, Pr(>F) =0.8369) during T0 or T1 (Fig. 7A). The calcification rates were, however, significantly reduced at T2 and T3 when corals were exposed to heat stress (PERMANOVA, T2: R2 = 0.309, Pr(>F) =0.029; T3: R2 = 0.449, Pr(>F) =0.0047) (Fig. 7A). The interaction between the factors, temperature and inoculation, was significant at the end of the experiment (PERMANOVA, T3: R2 = 0.141, Pr(>F) =0.0492), but no significant effect was determined for the inoculation itself (PERMANOVA, T3: R2 = 0.093, Pr(>F) =0.054).

For the respiration rates of the coral holobiont, no significant effects of temperature, inoculation, or the interaction of these factors (PERMANOVA, Pr(>F) >0.05) during T0 and T1 were observed, whereas temperature explained much of the differences between samples during T2 (PERMANOVA, R2 = 0.302, Pr(>F) =0.0353) and T3 (PERMANOVA, R2 = 0.387, Pr(>F) =0.0201). Inoculation did not contribute to the differences in respiration rates during any of the timepoints (Fig. S9A).

A similar pattern was observed in relation to the gross and net primary productivity of the corals, with neither of the two experimental factors driving differences in the first three timepoints (PERMANOVA, Pr(>F) >0.05), and temperature being the only factor contributing to significant differences between treatments at T3, with reduced values in the high-temperature groups (PERMANOVA, gross primary productivity: R2 = 0.544, Pr(>F) =0.0018; net primary productivity: R2 = 0.552, Pr(>F) =0.0007). Although the average values of primary productivity rates were higher in groups inoculated with the probiotics consortium by the end of the experiment, the inoculation treatment was not a significant factor explaining the variance in gross productivity (PERMANOVA, R2 = 0.064, Pr(>F) =0.1563) (Fig. S9B), although significant improvements were found in relation to their net primary productivity (PERMANOVA, R2 = 0.103, Pr(>F) =0.0490) (Fig. 7B).

## DISCUSSION

The presence of microorganisms in coral tissues has been widely reported (31, 32, 66, 67), and microbial therapies have been consistently linked to improvements in coral health and growth (18–20, 23–25, 68). However, the relationship between these two aspects is still unknown, which limits our understanding of whether those microorganisms found associated with the coral tissue are actually beneficial. Here, we expand our knowledge of the identity of coral-associated microbes and show the first evidence of the presence of validated BMCs inside coral host tissues, which supports the notion that their direct association with the host underpins their probiotic role.

After the use of BMCs as specific probiotics to improve coral health was proposed (12), Rosado and collaborators (23) proved this concept by using *P. damicornis*. The BMC consortium used at the time was initially selected based on genetic tests that targeted genes related to nitrogen cycling (*nirK* and *nifH*), as well as the *dmdA* gene involved in DMSP degradation and physiological assays for antioxidant activity. Further genomic analysis revealed other putative benefits to the coral host, such as genes related to the synthesis of vitamins, ectoines, and siderophores (37). This initial consortium, composed of isolates from coral and water samples, was validated as a BMC assemblage, being efficient in minimizing thermal bleaching and pathogen infection (23). Despite its promising use as a medicine to boost coral health and ensure their persistence through severe heatwaves and acute stress in laboratory trials (18, 19, 23), the actual location of these BMCs within the coral tissues was uncertain. As a consequence, it was unclear whether these BMCs were coral symbionts, consistently found associated with the coral tissue, or if they were transiently associated with the coral mucus or even being used as food (69).

In order to explore the effects of the BMCs in corals while also increasing our understanding of the identity of coral-associated microbes, we performed an experiment to investigate the localization of the probiotic strains used by Rosado and colleagues (23) even before their inoculation. We detected the two tested BMC strains *Halomonas* sp. and *Cobetia* sp. in the tissues of inoculated and uninoculated coral samples and found that they were significantly enriched by the probiotic inoculations. More specifically, our results show the first evidence that *Cobetia* sp. and *Halomonas* sp. can be inherently found inhabiting the coral ectodermis, adding to the scarce knowledge of coral-associated bacteria that was restricted to representatives of *Endozoicomonas* sp. (32), *Ralstonia* sp., Proteobacteria, and Actinobacteria (29, 30). Moreover, the relative abundance of *Halomonas* sp. and *Cobetia* sp. can increase through their inoculation as BMC, and such enrichment was correlated with the mitigation of the impacts of thermal stress in terms of *F*_v_/*F*_m_ rates and visual bleaching, as well as general improvements in productivity and calcification, which was not previously investigated (23).

The absence of major changes to the diversity or composition of the whole microbiome during heat exposure or inoculations indicates that during the experiment, *P. damicornis* may have acted as a “microbiome regulator” (70, 71). This means that the coral is able to maintain an overall stable microbiome even when facing heat stress, a finding similar to other studies where corals of *Pocillopora* genus maintained stable microbiomes when exposed to stressful conditions (70). The significant increases in *Halomonas* sp. and *Cobetia* sp. during the last time points of the experiment indicate, however, that certain strains were able to be enriched in corals and thus improve host health. The increase of the relative abundance of these two strains was more pronounced in BMC-treated corals at 26°C, indicating that, in this case, their enrichment was more successful when the corals are not heat-stressed. This finding differs from the first inoculation experiment reported by Rosado and collaborators (23), where *Cobetia* sp. was only enriched in heat-stressed corals when the overall microbiome had also changed. This contrast is likely a consequence of major differences in the experimental setup, temperature, coral colonies, water (artificial (23) vs natural seawater here), and inoculation regime. Nevertheless, colonization is not considered a requirement for probiotics (72), although in both cases, the inoculation of BMCs was correlated with improvements in coral health. Despite the temporary retention of applied probiotics or even the lack of coral colonization by a specific strain, even during its application, the inoculation of probiotics may promote a beneficial restructuring of the coral microbiome (18).

Specifically, similar to previous experiments, the inoculation with BMCs was able to mitigate the reduction of *F*_v_/*F*_m_ values in corals during heat stress (18, 23, 27), demonstrating possible protection of the photosystems of the endosymbiont algae. Higher average gross and net primary productivity values and calcification rates, which were not previously measured by Rosado and colleagues (23), were also detected in corals treated with the probiotic consortium. Due to the photosynthetic facilitation of calcification (73), higher calcification rates would be expected in samples that maintained higher *F*_v_/*F*_m_ and photosynthetic rates. As such, calcification values were higher in BMC-treated corals maintained at 26°C at the end of the experiment (T3). However, although BMC-treated corals had higher *F*_v_/*F*_m_ values and primary productivity rates than those treated with a saline solution, and higher calcification rate average values could be found in corals treated with BMCs, this did not translate into significantly higher calcification rates in heat-stressed corals at T3. This may be a consequence of the thermal stress and the energy being directed toward survivorship rather than growth. Probiotics in previous experiments have been able to increase calcification rates of healthy and heat-stressed corals (25, 26), an effect that could be useful for coral aquaculture practices used in active coral restoration to improve growth rates (74). Interestingly, the enzyme urease has been suggested to influence coral calcification (75), and genes encoding for this enzyme were found in the genomes of the *Halomonas* sp. and *Cobetia* sp. strains used here (37). However, the activity of the enzyme in these two bacteria still needs to be confirmed.

Temperature was also a key factor for respiration differences between experimental groups and was also seemingly unaffected by BMC inoculation. The lack of differences in holobiont respiration due to the inoculation of bacteria suggests that the input of heterotrophic bacteria was still not sufficient to drastically increase overall respiration rates, something that has been noted to occur in situations where the abundances of respiratory bacteria in corals becomes very high (76).

In this study, we expand the knowledge on the localization of bacteria within corals by showing that *Halomonas* sp. and *Cobetia* sp. are inherently associated with the coral tissue and that their relative abundance can be gradually increased by repeated inoculations. This observation argues against the possibility that the positive effects of the investigated BMC are due to factors unrelated to a direct symbiotic interaction, for example, that the host simply feeds on inoculated bacteria or that the bacteria change the water quality (77), although high abundances of beneficial bacteria in the water could still be possible with inoculation and could change the water quality in a manner that could benefit the studied corals. The presence of the bacteria inside both coral epidermal and gastrodermal tissues suggests an endosymbiotic status of the bacteria, which is recognized as a sign of intimate biological relations between microorganisms and cnidarians (28, 29, 78) or metazoans in general (79, 80). BMCs that are inherently associated with the coral tissue have also been suggested to be more likely to establish and promote long-term improvements to the holobiont (81). Additional studies are necessary to explore how long the enrichment of the tissue-associated BMCs *Halomonas* sp. and *Cobetia* sp. can be maintained, although studies on corals and different hosts (such as humans) indicate that the effect of probiotics and other microbial therapies is temporary and that the microbiome tends to return to its original assemblage after inoculations have ceased (18, 82, 83). Permanent (or even temporary) colonization is, however, not an absolute requirement for a probiotic to promote a beneficial impact on the host, as it may be caused by immune stimulation, epigenetic changes, and microbiome restructuring (18, 72, 84). Quick response and shifts that are correlated with environmental conditions (85), microbial succession and indirect effects (84, 86), and short-term colonization (87, 88) are, in fact, some of the main premises of the effectiveness of using probiotics (i.e., microbiomes are flexible (10, 89), as well as a safety advantage (90). It is important to note that probiotic-treated corals were still affected by palling, at significantly lower rates, and past experiments have also shown a faster recovery rate of probiotic-treated corals impacted by heat stress (15). Probiotics can be used as customized medicine when they are needed (e.g., during a heatwave) (91), minimizing mortality and retaining native colonies while other solutions are developed and applied. The microbiome assemblages may then return to their original state (although the health of the holobiont may continue to be boosted by epigenetic improvements) (84).

In summary, our findings suggest that *Halomonas* sp. and *Cobetia* sp. are coral endosymbionts able to inhabit both the coral gastrodermis and epidermis and therefore have the potential to have close biological interactions with their coral host. We also show that their enrichment, through active inoculation, correlates with the increase of the holobiont’s tolerance to heat stress and improved primary productivity and calcification rates. Altogether, this study was the first to locate specific strains of bacteria successfully used as probiotics in corals, working toward a better understanding of the association of beneficial organisms to coral hosts. Future studies may elucidate whether the enrichment of coral-associated BMCs also triggers long-term benefits for the host, through microbiome restructuring, immune responses, and/or epigenetic changes.

## Data Availability

All data needed to evaluate the conclusions in this paper are present in the paper and/or the supplemental material. All sequencing data are available at NCBI under the BioProject PRJNA956677 and BioSample IDs SAMN34227810–SAMN34227889. The scripts used to analyze physiological and microbial data can be found at Github: https://github.com/pedro-m-car/Cardoso_etal_BMCs.

## SUPPLEMENTAL MATERIAL

Supplemental Figures and Table msystems.00261-24-s0001.docx Figures S1-S9, Table S1, and captions for Tables S1-S4.

Table S1 msystems.00261-24-s0002.xlsx Target sequences of FISH probes.

Table S3 msystems.00261-24-s0003.xls Indicator zOTUs found on each experimental group throughout different time points.

Table S4 msystems.00261-24-s0004.xlsx zOTUs found as possible contaminants by the decontam package.

ASM does not own the copyrights to Supplemental Material that may be linked to, or accessed through, an article. The authors have granted ASM a non-exclusive, world-wide license to publish the Supplemental Material files. Please contact the corresponding author directly for reuse.
