# Supplementary material for: Localization and symbiotic status of probiotics in the coral holobiont
Source: mSystems. 2024 Apr 12;9(5):e00261-24. doi: 10.1128/msystems.00261-24 (PMC11097643; doi:10.1128/msystems.00261-24)
Supplement: Supplemental Figures and Table — Figures S1-S9, Table S1, and captions for Tables S1-S4. [file msystems.00261-24-s0001.docx]

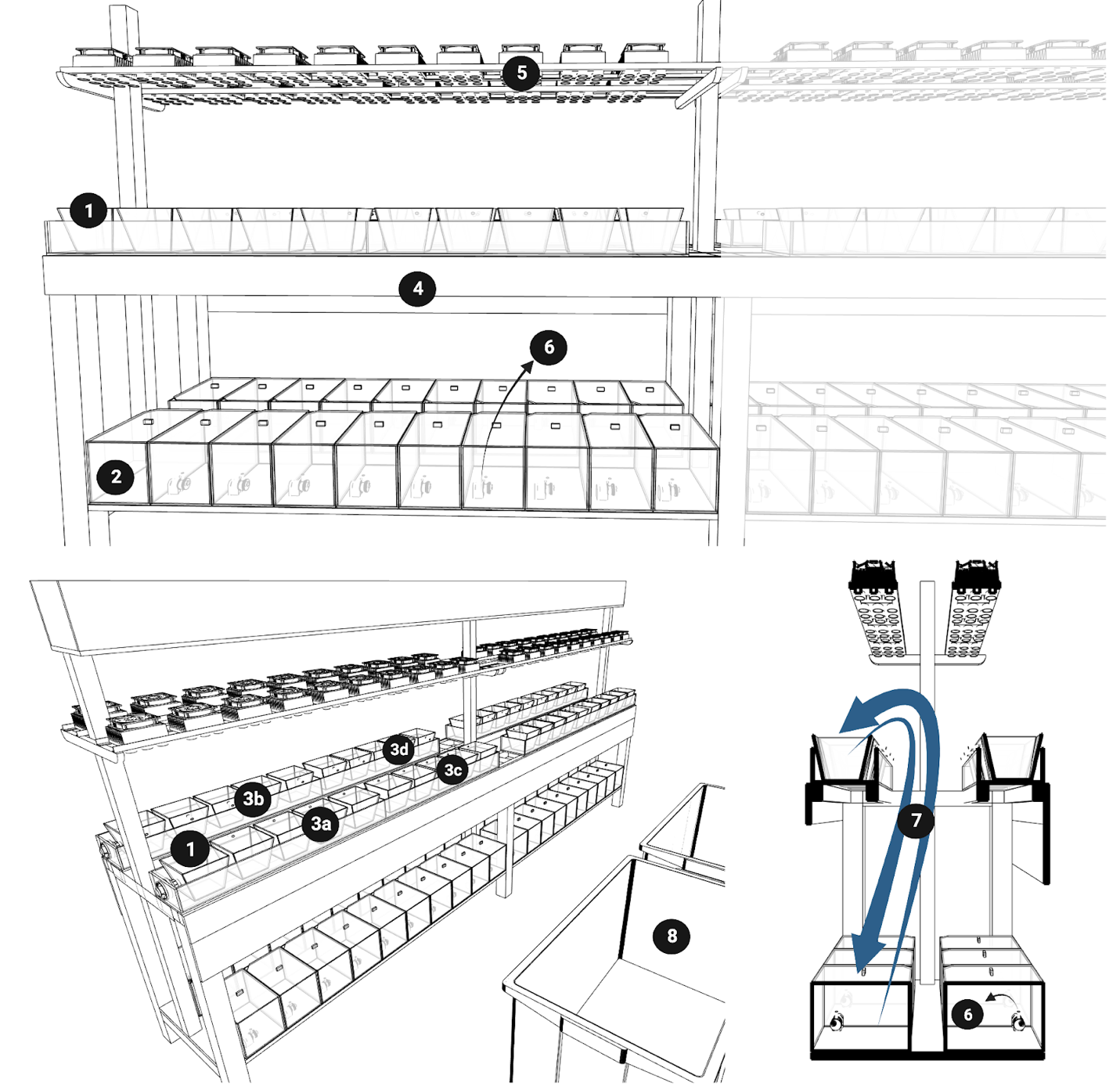


**Figure S1**: Schematic representation of the mesocosm set up for the experimental study on coral probiotics. Components include 20 experimental aquariums (1), each connected to their respective sump (2) to form an independent replication set. The experimental aquariums are immersed in four replicated water baths (3A, 3B, 3C, and 3D) with independent heating. The system is supported by a wooden base (4) and includes a dimmable LED light fixture with nine LED arrays (5). A recirculating pump (6) provides a water exchange rate of 170 L/h (7) between the sumps and the corresponding aquariums, and a cold water reservoir (8) is connected to each of the water baths for temperature control. The LED light fixture has a ratio of one white (6,000K) LED to three blue (465 nm) LEDs, each with a power of 3W and a 45° concentrator lens.


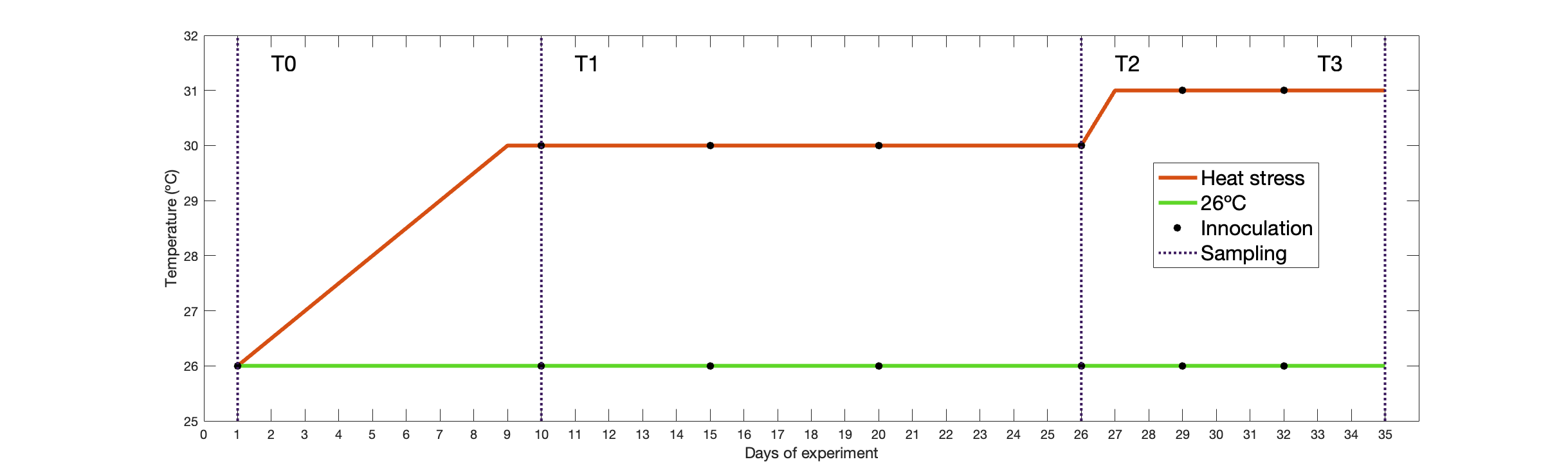


**Figure S2**: Temperature plot with general experiment schematic and inoculation and sample collection time points. The red line represents the temperature regime of the heat stress experimental groups, while the green line represents the temperature regime of the experimental groups kept at 26 °C. Black spots indicate days in which inoculation took place, and dotted lines represent days in which coral samples were collected.


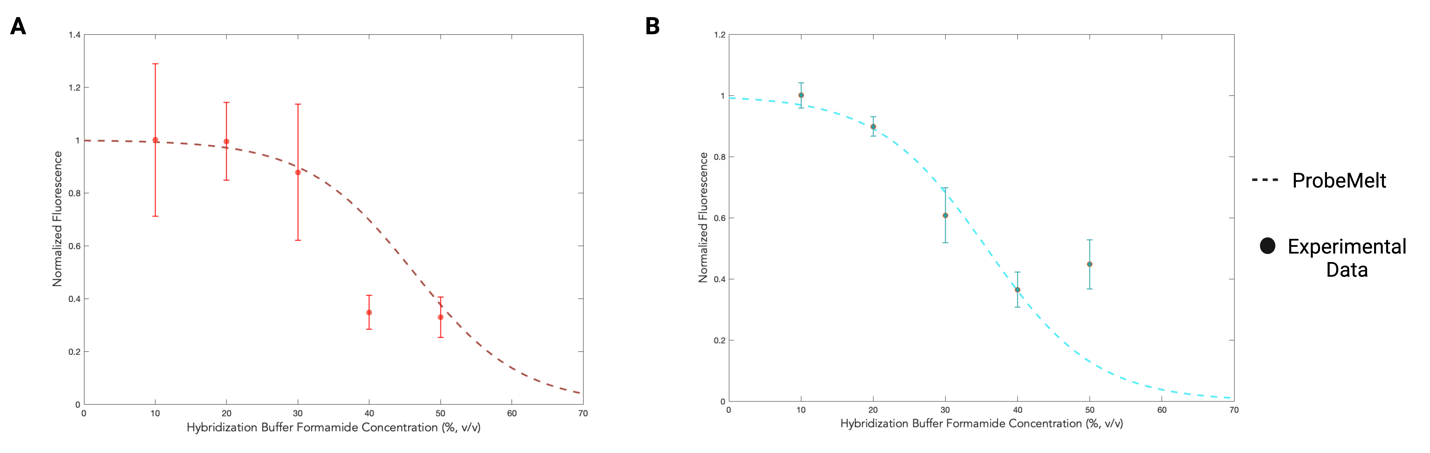


**Figure S3:** Denaturation curves comparing experimental to theoretical data related to customized FISH probes. The COB1268 (A) and HAL847(B) probes were tested in hybridization reactions through flow cytometry with varying formamide levels. Points show median fluorescence levels normalized by the maximum value obtained during the test. Dotted lines illustrate the theoretical values predicted by the ProbeMelt software. Error bars represent the standard deviations of three hybridization replicates.


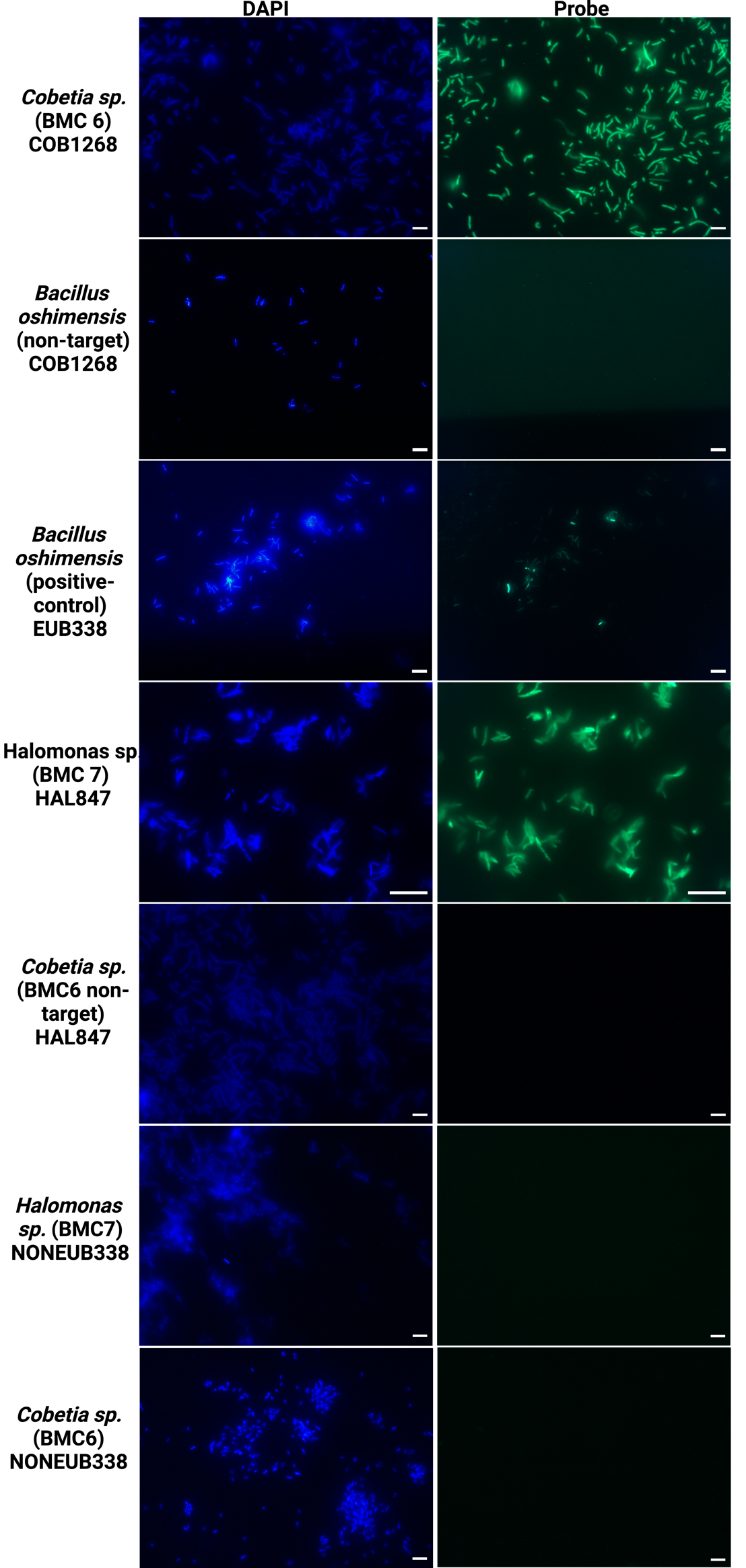


**Figure S4:** FISH images of probe specificity tests made with pure cultures of *Halomonas sp*. (BMC 7) and *Cobetia sp.* (BMC 6) and *Bacillus oshimensis* (M24) labeled with specific probes designed to target each one of these strains. Cyan = DAPI; Green = specific probe. Scale Bars = 10 μm.


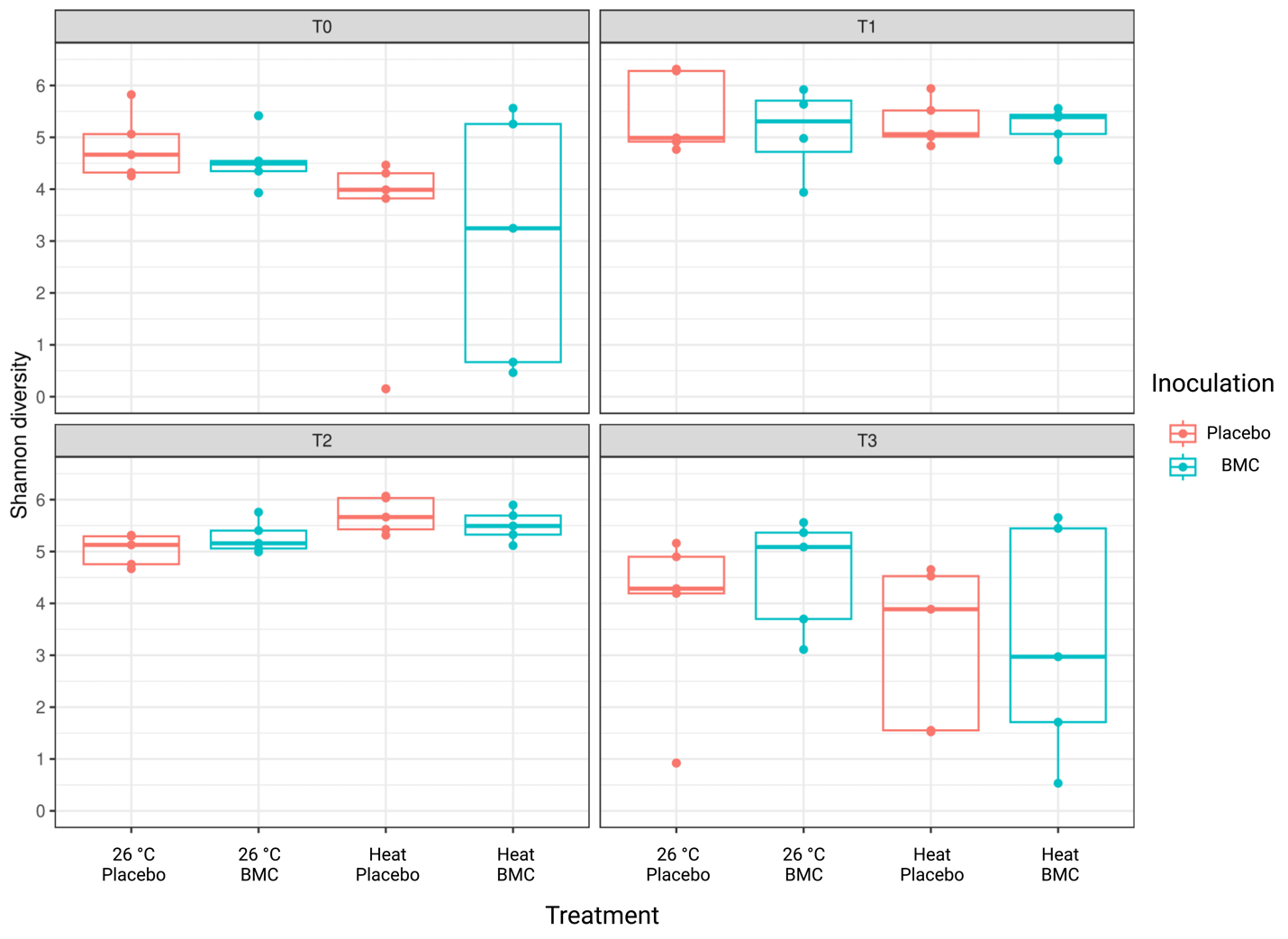


**Figure S5:** Alpha diversity based on the Shannon index of microbial communities associated with coral fragments treated with the BMC probiotic consortium and a saline solution during the four time-points of the experiment: T0 (day 1), T1 (day 10), T2 (day 26) and T3 (day 35). Box edges represent quartiles, while middle horizontal bars represent median values.


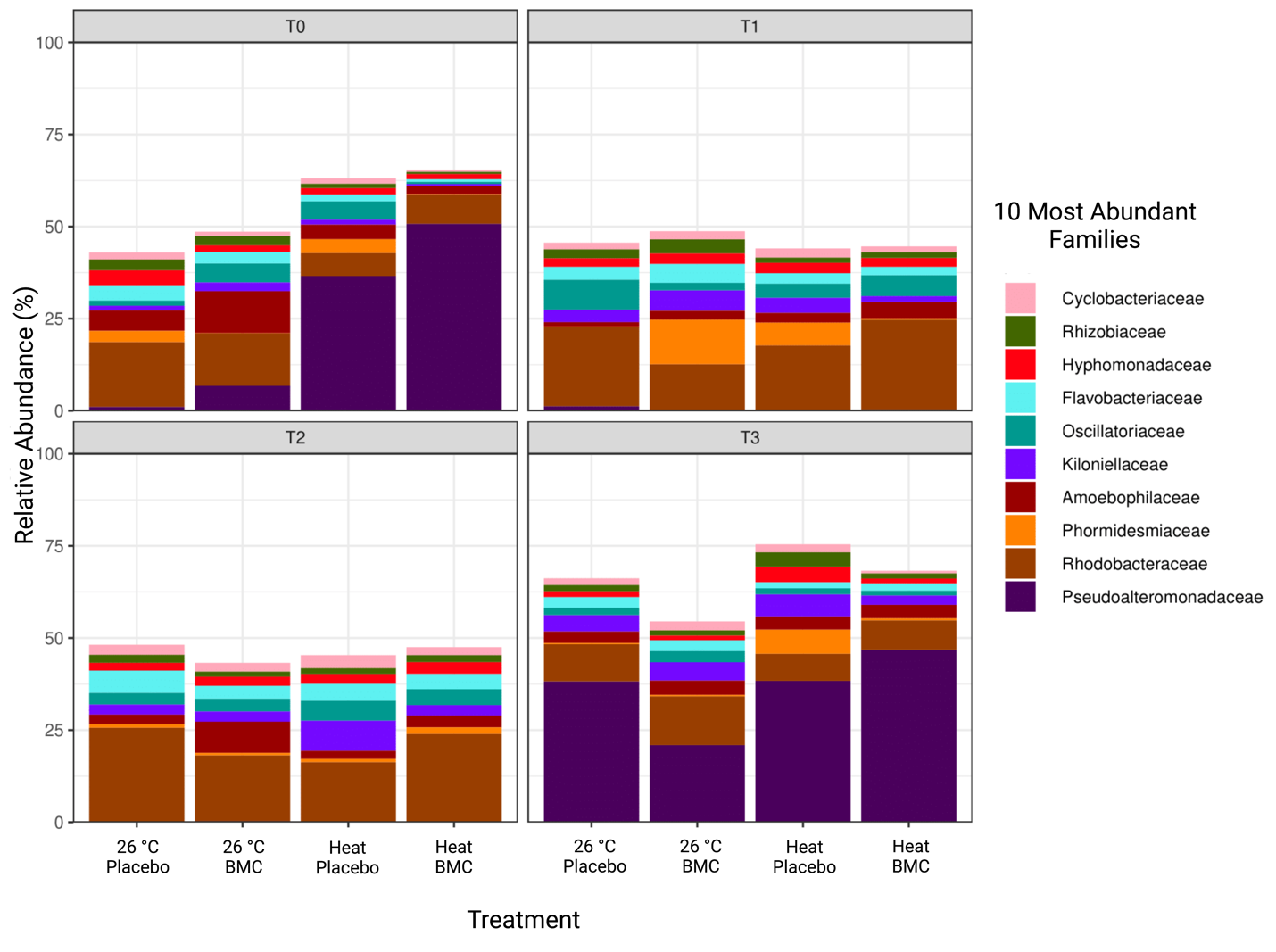


**Figure S6:** Relative abundance of the ten most abundant families of microbial communities associated with coral fragments treated with the BMC probiotic consortium and a saline solution during the four time-points of the experiment: T0 (day 1), T1 (day 10), T2 (day 26) and T3 (day 35), found by 16S gene sequencing.


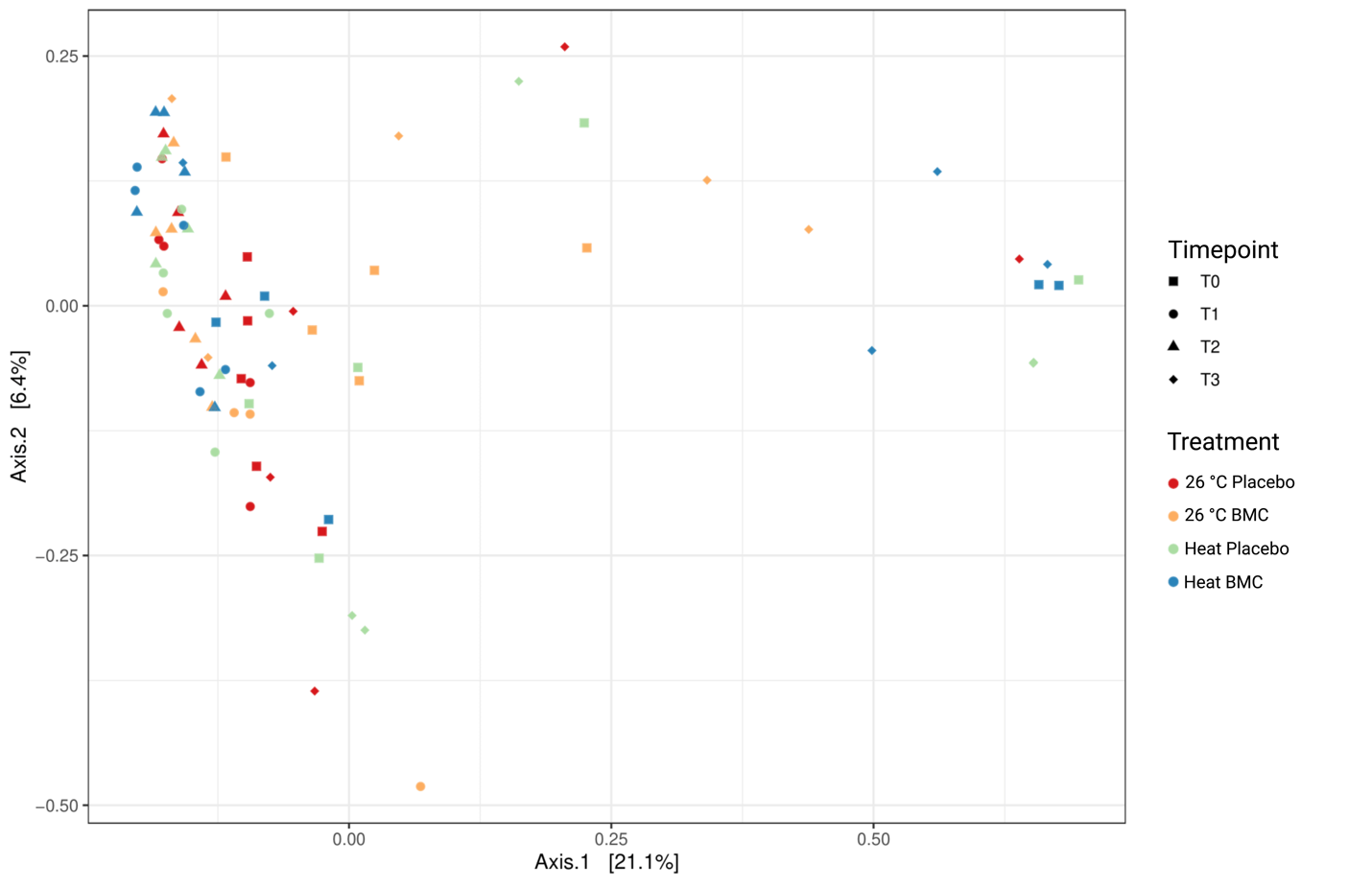


**Figure S7:** PCoA based on Bray-Curtis dissimilarities of zOTU-level data for the microbial communities associated with coral fragments treated with the BMC probiotic consortium and a saline solution during the four time-points of the experiment: T0 (day 1), T1 (day 10), T2 (day 26) and T3 (day 35) obtained from 16S gene sequencing.


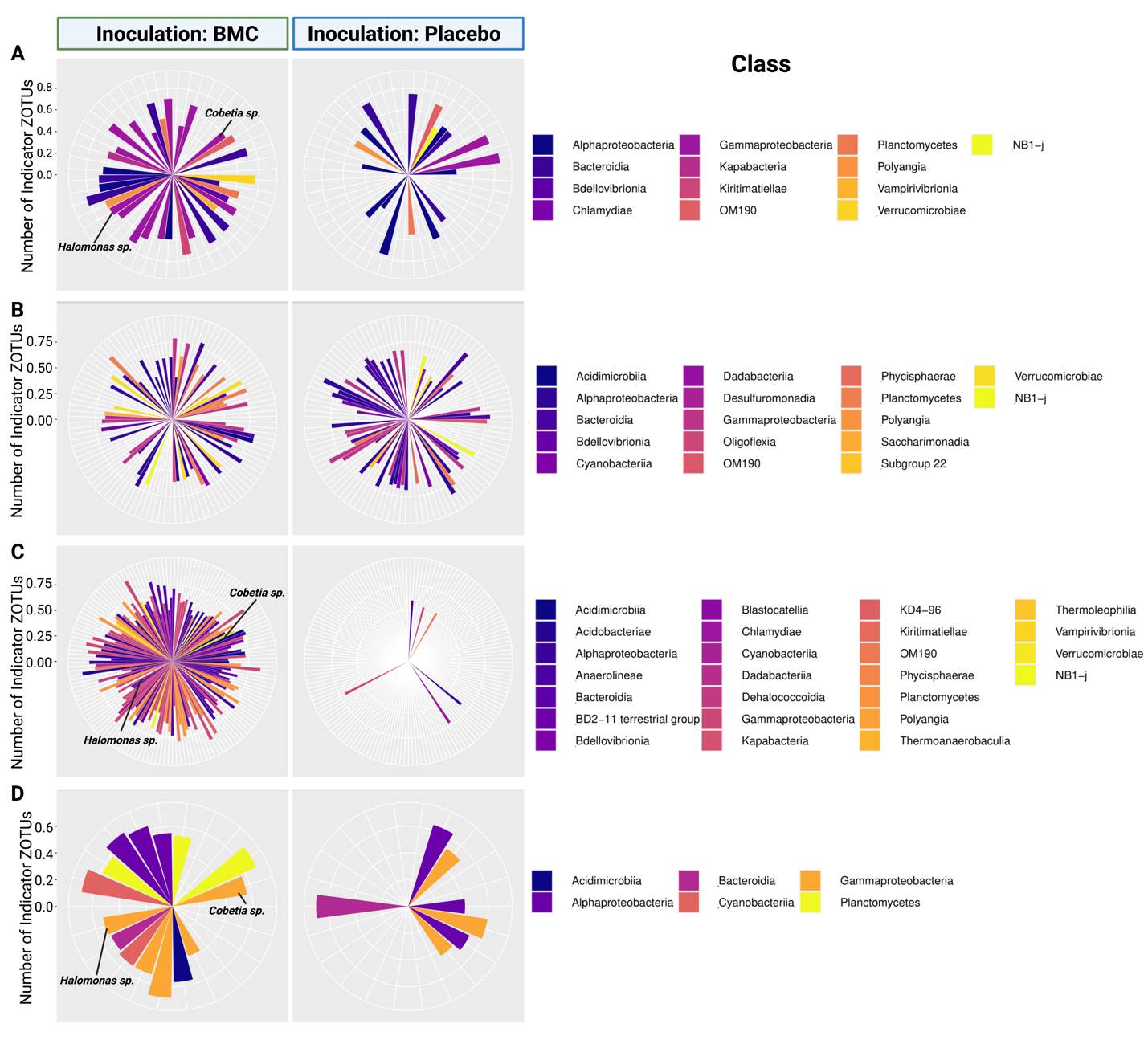


**Figure S8:** Indicator zOTUs identified by comparing BMC inoculated corals to fragments from the placebo group: (A) T2 (26 days) under 26 °C, (B) T2 (26 days) under heat treatment, (C) T3 (35 days) under 26 °C, and (D) T3 (35 days) under heat treatment. Each bar represents a single indicator zOTU (p < 0.05) and bar colors represent the class of each zOTU. The y axis represents the indicator value of each zOTU in the samples of each group. zOTUs with identical sequences to the strains inoculated as probiotics are indicated by text.


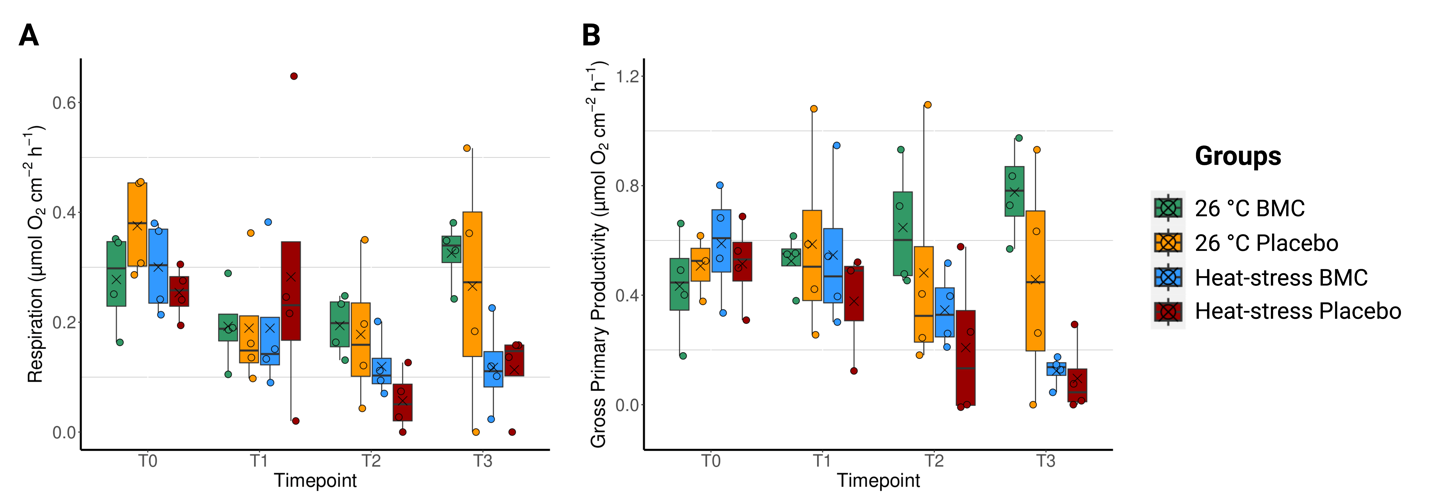


**Figure S9:** Respiration (A) and gross primary-productivity (B) values of *Pocillopora damicornis* fragments during the four timepoints of the experiment: T0 (day 1), T1 (day 10), T2 (day 26) and T3 (day 35), n = 4. Jitter points represent values from individual replicates, crosses represent mean values of each group, box edges represent quartiles, while middle horizontal bars represent median values.

**Table S1:** Target sequences of FISH probes used in this study of target and non-target bacteria.


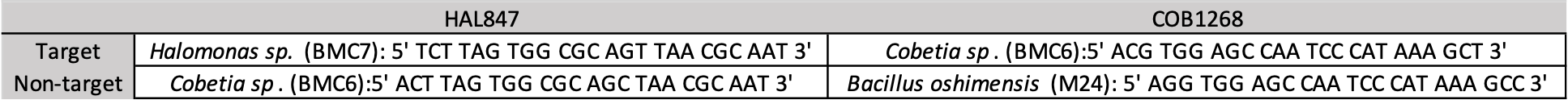


**Table S2**: Spreadsheet of all zOTUS found in study with their respective taxonomic classification derived from the Silva database.

**Table S3:** Spreadsheet listing the indicator zOTUs found on each experimental group throughout different time points.

**Table S4:** Spreadsheet listing the zOTUs found as possible contaminants by the decontam package.
